# *GA20ox* Family Genes Mediate Gibberellin and Auxin Crosstalk in Moso bamboo (*Phyllostachys edulis*)

**DOI:** 10.3390/plants12152842

**Published:** 2023-08-01

**Authors:** Yucong Bai, Yali Xie, Miaomiao Cai, Jutang Jiang, Chongyang Wu, Huifang Zheng, Jian Gao

**Affiliations:** Key Laboratory of National Forestry and Grassland Administration, Beijing for Bamboo & Rattan Science and Technology, International Center for Bamboo and Rattan, Beijing 100102, China; bai.yucong@icbr.ac.cn (Y.B.); xieyali@icbr.ac.cn (Y.X.); cmm@icbr.ac.cn (M.C.); jiangjutang@icbr.ac.cn (J.J.); wcy@icbr.ac.cn (C.W.); zhenghuifang@icbr.ac.cn (H.Z.)

**Keywords:** Moso bamboo, *GA20ox* gene family, gibberellin, auxin crosstalk

## Abstract

Moso bamboo (*Phyllostachys edulis*) is one of the fastest growing plants. Gibberellin (GA) is a key phytohormone regulating growth, but there are few studies on the growth of Moso bamboo regulated by GA. The *gibberellin 20 oxidase* (*GA20ox*) gene family was targeted in this study. Chromosomal distribution and collinearity analysis identified 10 *GA20ox* genes evenly distributed on chromosomes, and the family genes were relatively conservative in evolution. The genetic relationship of *GA20ox* genes had been confirmed to be closest in different genera of plants in a phylogenetic and selective pressure analysis between Moso bamboo and rice. About 1/3 *GA20ox* genes experienced positive selective pressure with segmental duplication being the main driver of gene family expansion. Analysis of expression patterns revealed that only six *PheGA20ox* genes were expressed in different organs of shoot development and flowers, that there was redundancy in gene function. Underground organs were not the main site of GA synthesis in Moso bamboo, and floral organs are involved in the GA biosynthesis process. The auxin signaling factor PheARF47 was located upstream of *PheGA20ox3* and *PheGA20ox6* genes, where PheARF47 regulated *PheGA20ox3* through cis-P box elements and cis-AuxRR elements, based on the result that promoter analysis combined with yeast one-hybrid and dual luciferase detection analysis identified. Overall, we identified the evolutionary pattern of *PheGA20ox* genes in Moso bamboo and the possible major synthesis sites of GA, screened for key genes in the crosstalk between auxin and GA, and laid the foundation for further exploration of the synergistic regulation of growth by GA and auxin in Moso bamboo.

## 1. Introduction

Gibberellin (GA) is an essential phytohormone in plant growth and development and has a significant regulatory role in seed germination, stem elongation, leaf development, flower-forming transformation, and flower development [1,2,3,4]. GA synthesis is a complex pathway and although many catalytic enzymes are involved in this process, gibberellin 20 oxidase (GA20ox) is an important rate-limiting enzyme involved in GA synthesis [5,6,7]. *GA20ox-2* is considered to be the ‘Green Revolution’ gene, and its mutation has resulted in dwarf varieties of rice, which has greatly improved rice yields [8,9,10].

GA20ox belongs to soluble dioxygenases (2-oxoglutarate-dependent dioxygenase (2ODD)). GA passes through the plastid and endoplasmic reticulum to form gibberellin 12 (GA12) from geranylgeranyl diphosphate (GGDP), and then GA20ox converts GA12 to gibberellin 4 (GA4) in the cytoplasm [11,12]. When *GA20ox* is mutated or overexpressed, GA synthesis is significantly affected, which in turn affects plant height or other growth traits [13,14]. Therefore, *GA20ox* is a key target for genetic engineering and manipulation of economically important traits controlled by GA.

Phytohormone crosstalk by GA has been better studied in many plants over the last few decades, particularly in auxin and GA. The biological functions of auxin and GA overlap and interact in a number of aspects, including the regulation of root growth and organ expansion during plant development [15,16]. The interaction between auxin and GA is predominantly mediated by the auxin signaling proteins auxin/indole-3-acetic acid (Aux/IAA) and auxin response factor (ARF) [17]. Aux/IAA and ARF not only regulate GA metabolizing enzymes, including GA20ox, gibberellin 3 oxidase (GA3ox), and gibberellin 2 oxidase (GA2ox), but also negatively regulate GA signaling DELLA proteins. In Arabidopsis, Aux/IAA and ARF proteins can directly regulate *AtGA20ox* and *AtGA2ox* expression, while GA can directly impair the phenotype of certain functional *Aux/IAA* genes [18]. In poplar, *GA20ox* overexpression regulates biomass accumulation and lateral root formation by crosstalk with auxin and abscisic acid [19]. In tomato, the SlARF7-SlIAA9 interaction blocks GA biosynthesis and auxin metabolism by repressing *GA20ox1/GA3ox1* and *GH3.2* expression and prevents transcriptional activation of genes that promote fruit formation by forming the SlARF7-SlIAA9 and SlARF7-SlDELLA complexes [20]. In contrast, the application of GA to tomatoes that are not sensitive to auxin induces auxin signaling factor ARF expression and promote cell expansion [21]. The presence of ARF and GA has the effect of limiting fruit cell division and promotes cell expansion. The ability of ARF to interact with many genes is critical for promoting the auxin–GA crosstalk [22].

Moso bamboo (*Phyllostachys edulis*) is recorded in the *Guinness Book of World Records* as one of the fastest growing plants on earth, at up to 1 m/d. Moso bamboo shoots are edible and the culms can be processed for bamboo timber, which has a high ecological and economic value [23,24,25]. Phytohormones play a crucial role in the fast growth of bamboo shoots, and GA and auxin directly affect the internode length [26,27]. However, the phytohormone signaling crosstalk between GA and auxin in Moso bamboo is still unclear. Studies on *GA20ox*, a key gene involved in GA synthesis, has also been limited to traditional gene family analysis, and systematic identification and characterization of *GA20ox* in terms of evolutionary relationships and related regulatory signaling pathways are still lacking [28,29]. On this basis, we explored the evolutionary process of the gene family from the evolutionary relationship of the *GA20ox* gene family and identified the target genes by expression pattern analysis. Using yeast one-hybrid and dual luciferase techniques, we demonstrated that ARF47 could regulate the growth and development of Moso bamboo by binding the promoters of *GA20ox3* and *GA20ox6*. It was revealed that there is an interaction between GA and auxin to jointly regulate plant development.

## 2. Results

### 2.1. Analysis of the Chromosomal Distribution and Evolutionary Patterns of the GA20ox Gene Family

**Figure 1:**
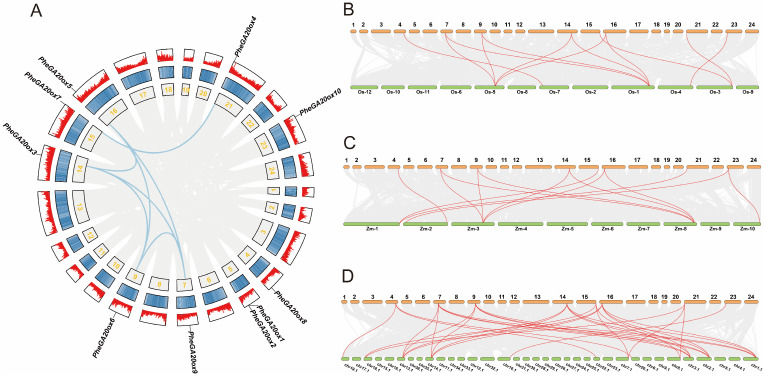
Chromosomal distribution and collinearity analysis of the *GA20ox* gene family of genes in Moso bamboo. (**A**) The relationships among the chromosomes of the Moso bamboo *GA20ox* gene family members. (**B**–**D**) Analysis of collinearity relationships between Moso bamboo and rice, Moso bamboo and maize, Moso bamboo and Ma bamboo.

With the release of the latest version of the Moso bamboo genome, gene family identification and structural analysis of the *GA20ox* gene family had been carried out by researchers but had not been explored in depth. In this study, we first identified the chromosomal distribution of the *GA20ox* gene family after analyzing the physicochemical properties (Figure 1A, Table S1). The analysis result showed that the 10 genes of the *PheGA20ox* gene family in Moso bamboo were localized on 9 of the 24 chromosomes. In addition to *PheGA20ox1* and *PheGA20ox2* co-distributed on chromosome 5, the other eight *PheGA20ox* genes were evenly distributed on chromosomes 4, 7, 9, 14, 15, 16, 21, and 23. A total of seven *PheGA20ox* gene pairs were distributed on different chromosomes of the genome, suggesting that there were chromosomal segmental replication events during the formation of the *PheGA20ox* gene family, rather than tandem replication. Chromosomal segmental replication was the main driver of gene family expansion.

To further analyze the evolutionary patterns of the *PheGA20ox* gene family, in addition to intraspecies collinearity analysis of the *PheGA20ox* gene family, we also constructed a collinearity map among species at the genome-wide level, including Moso bamboo, rice, maize, and Ma bamboo (Figure 1B, Table S2). Among them, rice and maize belong to the same monocotyledonous group as Moso bamboo, and Ma bamboo was a species under Bambusoideae, which was more intensively studied. The analysis revealed a total of eight *PheGA20ox* genes in collinearity blocks with rice (5), maize (6), and Ma bamboo (12), with 12, 13, and 30 gene pairs between Moso bamboo and rice, maize, and Ma bamboo, respectively. Most of the *PheGA20ox* genes were homologous, and eight *PheGA20ox* genes emerged and were stable during the early evolutionary stages. The gene family was evolutionarily conserved.

### 2.2. Phylogenetic Tree Construction and Selection Pressure Analysis of the GA20ox Gene Family

**Figure 2:**
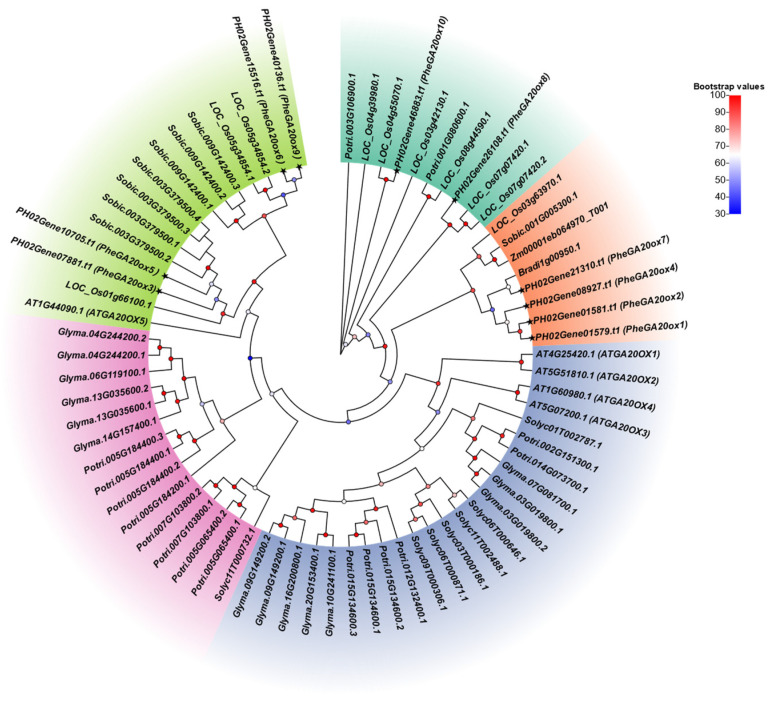
Phylogenetic tree of Moso bamboo *GA20ox* gene family. A total of 72 *GA20ox* genes from nine species are included. Specifically, these include: *Oryza sativa* (10), *Arabidopsis thaliana* (5), *Sorghum bicolor* (8), *Zea mays* (1), *Glycine max* (14), *Populus trichocarpa* (16), *Brachypodium distachyon* (1), *Solanum lycopersicum* (7), and *Phyllostachys edulis* (10).

To investigate the phylogenetic patterns of the *GA20ox* gene family in Moso bamboo, a phylogenetic tree was constructed by maximum likelihood method for 72 *GA20ox* genes selected from nine species, including monocotyledonous and dicotyledonous plants, herbs and woody plants, and annuals and perennial plants, namely, *Oryza sativa* (10), *Arabidopsis thaliana* (5), *Sorghum bicolor* (8), *Zea mays* (1), *Glycine max* (14), *Populus trichocarpa* (16), *Brachypodium distachyon* (1), *Solanum lycopersicum* (7), and *Phyllostachys edulis* (10) (Figure 2). Five subgroups were identified based on the topology of the phylogenetic tree, and the *PheGA20ox* genes were distributed in three subgroups (n = 4, 4, 2). Two of the three subgroups contained both monocotyledons and dicotyledons, but the *PheGA20ox* gene family belonged to the same or adjacent branches as the monocotyledons. Among them, *PheGA20ox* genes such as *PheGA20ox3*, *PheGA20ox6*, *PheGA20ox8*, *PheGA20ox9,* and *PheGA20ox10* were more closely related to rice, predicting that these genes were more similar to rice in terms of potential biological functions.

**Figure 3:**
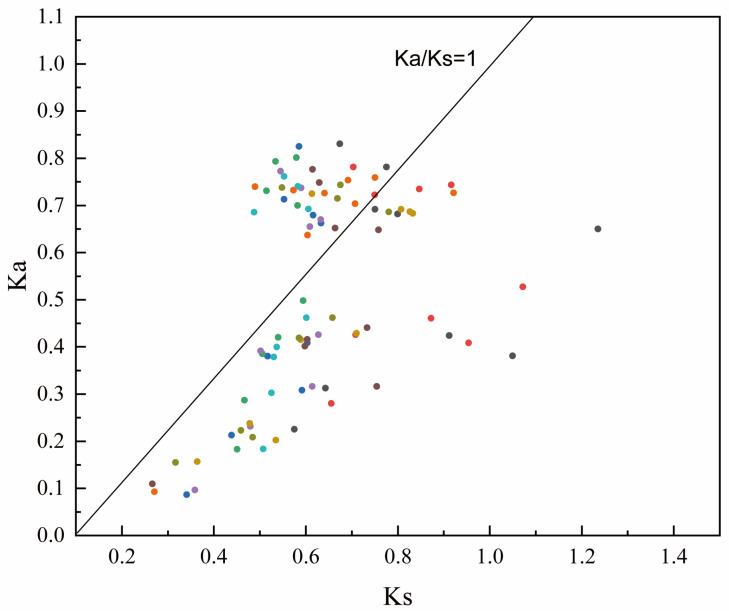
Analysis of selection pressure on homologous genes of the Moso bamboo and rice *GA20ox* families. Different colors represent different Moso bamboo *GA20ox* genes.

Next, we calculated the ratio of Ka/Ks between the *GA20ox* genes of Moso bamboo and rice to further investigate the evolutionary pressure on the *GA20ox* gene family (Figure 3, Table S3). In all, 66% of the gene pairs were Ka/Ks < 1, indicating that they were undergoing purifying selection, while 10% of the gene pairs were Ka/Ks = 1, indicating that they were undergoing neutral selection. Surprisingly, positive selection pressure (Ka/Ks > 1) was present in 24% of *GA20ox* gene pairs, suggesting the presence of gene family expansion events in *GA20ox*, with chromosomal segmental duplication being the main driver of gene family expansion, as shown in Figure 1.

### 2.3. Analysis of the Expression Pattern of the GA20ox Gene Family

**Figure 4:**
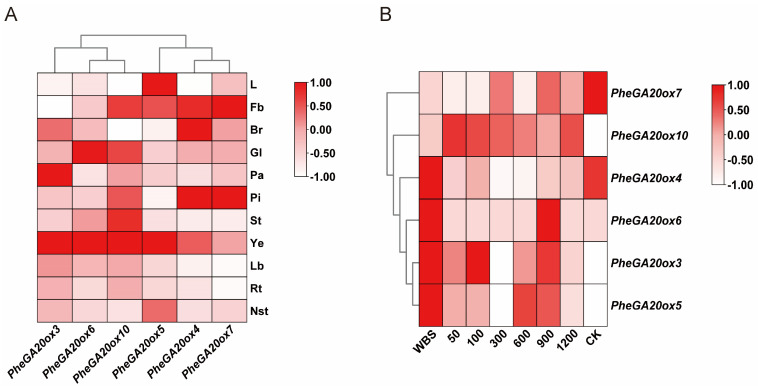
Analysis of *GA20ox* gene expression patterns based on transcriptome data. (**A**) Expression patterns of *GA20ox* family genes in different organs. L: leaf, Fb: flower bud, Br: bract, Gl: glume, Pa: palea, Pi: pistil, St: stamen, Ye: young embryo, Lb: Lateral bud, Rt: Rhizome tip, Nst: New shoot tip. (**B**) Expression patterns of *GA20ox* family genes in Moso bamboo shoots during different developmental stages. WBS: winter bamboo shoots, 50–1200: bamboo shoot lengths (in cm), and CK represents bamboo plants with spreading leaves. The color scale represents the z-scores.

To investigate the biological functions of the *PheGA20ox* gene family, we analyzed its expression pattern in different organs and developmental stages of Moso bamboo shoots based on transcriptomic data (Figure 4). The analysis showed that only six *PheGA20ox* gene family members were differentially expressed in different organs (L (leaf), Fb (flower bud), Br (bract), Gl (glume), Pa (palea), Pi (pistil), St (stamen), Ye (young embryo), Lb (Lateral bud), Rt (Rhizome tip), Nst (New shoot tip)) of Moso bamboo. Therefore, further analysis of the six differentially expressed genes revealed that different *PheGA20ox* genes were expressed in different patterns (Figure 4A). *PheGA20ox3*, *PheGA20ox6,* and *PheGA20ox10* had the highest expression levels in the young embryo, *PheGA20ox5* was highly expressed in the leaves, *PheGA20ox4* had a high expression pattern in the pistil, and *PheGA20ox7* had the highest transcript levels in the flower bud and pistil. However, unlike other plants, the *PheGA20ox* gene family was not expressed in the roots or was expressed at low levels, suggesting that it may have a weak biological function in underground organs. The floral organs were involved in the GA biosynthesis process.

The expression levels at different developmental stages of the bamboo shoots (WBS (winter bamboo shoots), 50, 100, 300, 600, 900, and 1200 cm bamboo shoot heights, and CK (bamboo plants with spreading leaves)) showed that *PheGA20ox3*, *PheGA20ox4,* and *PheGA20ox5* were expressed at higher levels in winter bamboo shoots, and *PheGA20ox6* was highly expressed in winter bamboo shoots and 900 cm bamboo shoots, where all four genes were weakly expressed in 300 cm bamboo shoots. However, *PheGA20ox7* and *PheGA20ox10* expression levels showed the opposite trend, with *PheGA20ox7* being expressed at the highest level in CK and *PheGA20ox10* being barely expressed in CK (Figure 4B). Different *PheGA20ox* genes played different roles in different developmental stages of bamboo shoots and may be involved in various stages of Moso bamboo growth and development.

### 2.4. Analysis of Phytohormone Response Elements in the Promoter Region of GA20ox Family Genes

**Figure 5:**
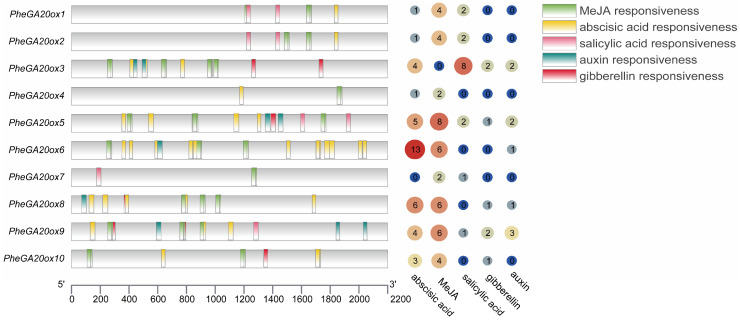
Analysis of the promoters of the *GA20ox* family genes in Moso bamboo. Numbers represent the number of relevant cis-acting elements.

Previous studies have shown that GA regulates plant growth and development through phytohormone crosstalk, so we analyzed the phytohormone-responsive elements in the 2000 bp region upstream of the transcriptional start of the *GA20ox* genes (Figure 5, Table S4). The *GA20ox* family genes had 38, 50, 8, 7, and 9 elements responding to abscisic acid, methyl jasmonate (MeJA), salicylic acid, GA, and auxin, respectively, accounting for 34%, 45%, 7%, 6%, and 8%. Of the *GA20ox* genes, 30% had four phytohormone response elements, 40% of the *GA20ox* genes had three phytohormone response elements, and the remaining 20% of the *GA20ox* genes had two phytohormone response elements. This predicted that phytohormones may regulate *PheGA20ox* to influence the growth and development of Moso bamboo. Notably, *PheGA20ox3*, *PheGA20ox5,* and *PheGA20ox6* had more salicylic acid (8), MeJA (8), and abscisic acid (13) hormone response elements, respectively, and it was speculated that these three genes may play a key role in response to other phytohormones.

### 2.5. Analysis of Upstream Regulators of PheGA20ox3 and PheGA20ox6

**Figure 6:**
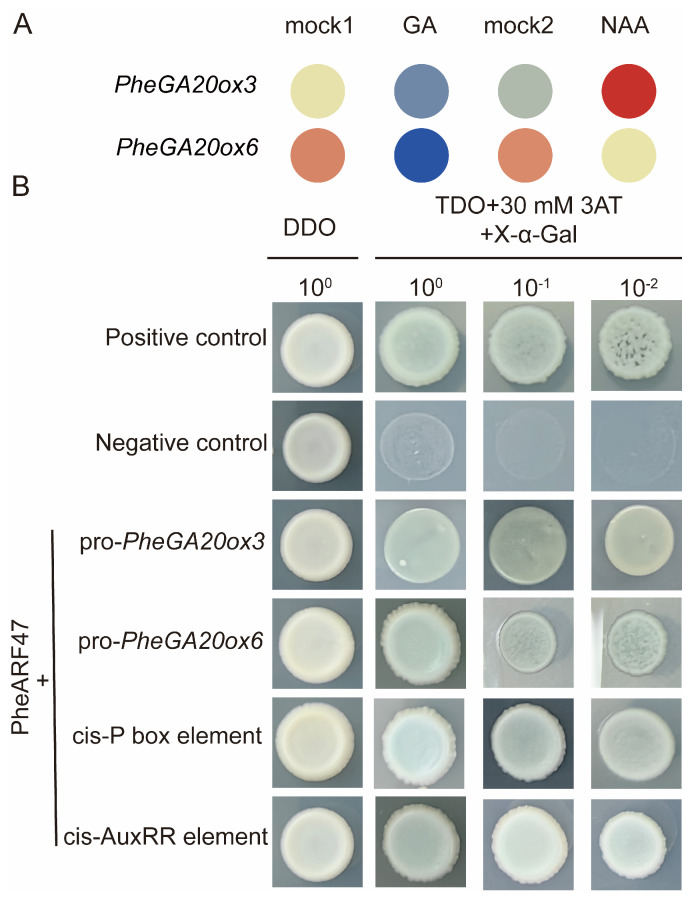
Analysis of the phytohormone response pattern and upstream regulators of the *GA20ox* family genes in Moso bamboo. (**A**) Analysis of exogenous phytohormone response patterns of *PheGA20ox3* and *PheGA20ox6*. mock1, GA, mock2, NAA represent the treatments of 1-month-old Moso bamboo seedlings with water, gibberellin 3 (GA3), water, and naphthalene acetic acid (NAA), respectively. (**B**) Yeast one-hybrid assay. Positive control is pGAD T7 53 + pHIS2 53 and negative control is pHIS2 53 + pGADT7 Rec2. pro-*PheGA20ox3* and pro-*PheGA20ox6* represent the *PheGA20ox* gene promoter region and cis-P box element, and cis-AuxRR element represents the P box element sequence (CCTTTTTG) and AuxRR element (GGTCCAT) repeated three times. All promoter sequences are linked to the pHIS2 vector and PheARF47 to the pGADT7 vector.

Based on the transcriptome data analysis (one-month-old seedlings of Moso bamboo treated by GA3 and naphthalene acetic acid), only *PheGA20ox3* (*PH02Gene07881.t1*) and *PheGA20ox6* (*PH02Gene15516.t1*) were found to respond to exogenous GA and naphthalene acetic acid (NAA) treatments (Figure 6A). *PheGA20ox3* was upregulated under NAA treatment and downregulated under GA treatment, while *PheGA20ox6* was downregulated under both NAA and GA treatments. Combined with the results of the phytohormone response element analysis in the promoter region, we selected the *PheGA20ox3* and *PheGA20ox6* to explore possible phytohormone regulators upstream of them by yeast one-hybrid (Figure 6B). We cloned the promoter regions of *PheGA20ox3* and *PheGA20ox6* and screened the yeast library of Moso bamboo shoot to obtain PheARF47 (*PH02Gene44368.t1*) (Table S5), the upstream transcription factor that co-regulates *PheGA20ox3* and *PheGA20ox6.* Then the CDS region of the *PheARF47* gene was cloned, and the binding of *PheARF47* to the promoters of *PheGA20ox3* and *PheGA20ox6* was confirmed again by yeast one-hybrid. We also found that PheARF47 could bind the cis-P box element (CCTTTTTG), a GA-binding element, and the cis-AuxRR element (GGTCCAT), an auxin-binding element.

### 2.6. Analysis of the PheARF47-Regulated PheGA20ox Promoter Region

**Figure 7:**
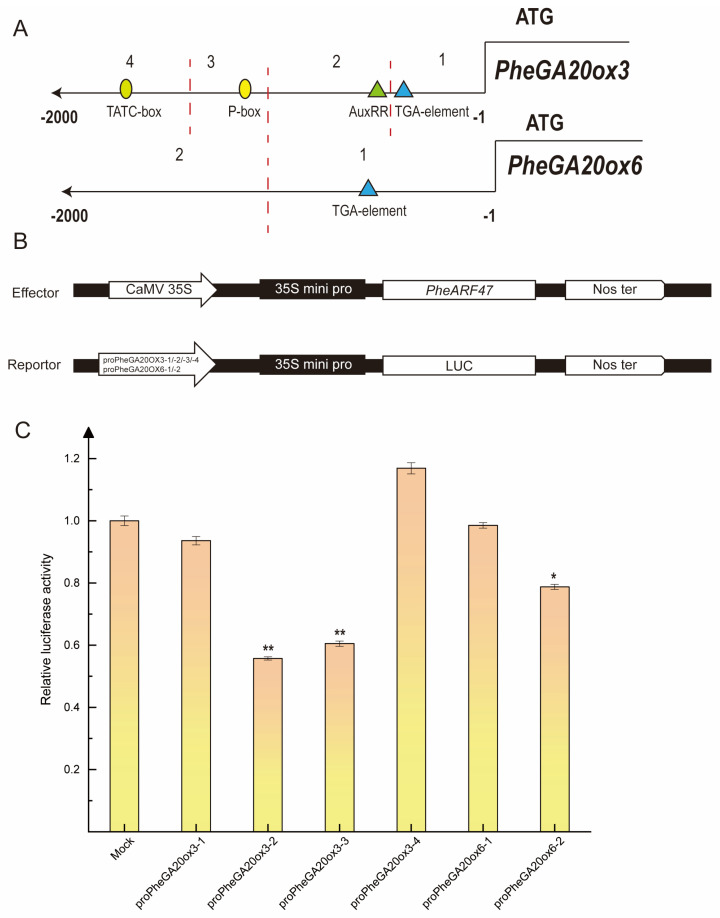
Analysis of upstream regulatory elements of the *PheGA20ox3* and *PheGA20ox6* genes. (**A**) Schematic diagram of promoter segmentation based on auxin and GA response elements in the promoter region. (**B**) Schematic diagram of the dual luciferase vector construction. The CDS region of the *PheARF47* gene was ligated into the pGreenII 62-SK vector, and the promoter region segments of the *PheGA20ox3* and *PheGA20ox6* genes were introduced into the pGreenII 0800-LUC vector. (**C**) The analysis results of relative fluorescence activity. The luminescence ratio of firefly LUC to Renilla LUC was determined according to a dual luciferase reporter system. Experiments were repeated at least three times and results are expressed as mean ± standard deviation. Statistics were determined by using a *t* test (** represents *p* < 0.01, * represents *p* < 0.05).

To further validate that PheARF47 binds specific regions of the *PheGA20ox3* and *PheGA20ox6* gene promoters, a dual luciferase reporter system was constructed and transiently expressed in tobacco (Figure 7B). Because PheARF47 probably functions by binding the binding elements of auxin and GA, the *PheGA20ox3* gene promoter was distinguished into four segments including the auxin-binding elements (AuxRR, TGA element) and the GA-binding elements (TATC box, P box). The *PheGA20ox6* promoter region was divided into two segments, one of which contained an auxin-binding element (TGA element), and the other did not contain auxin or a GA-binding element (Figure 7A). The results showed that PheARF47 could inhibit the expression of PheGA20ox3-2, PheGA20ox3-3, and PheGA20ox6-2, which further confirmed that PheARF47 could bind the promoters of *PheGA20ox3* and *PheGA20ox6* (Figure 7C). Overall, PheARF47 could regulate *PheGA20ox3* gene expression through the P box element and the AuxRR element.

## 3. Discussion

GA can facilitate the transition from normal to rapid growth by promoting cell elongation and cell division in Moso bamboo shoots [27,30]. GA20ox is a key enzyme in GA synthesis and functions as a rate-limiting enzyme in GA synthesis. It is encoded by a small gene family and at this stage in Arabidopsis, rice, and maize species, and 5, 10, and 1 family members have been identified [31,32,33]. There are 10 *GA20ox* genes in Moso bamboo, but only 6 *GA20ox* genes responded to Moso bamboo shoot-bamboo growth and development, implying a possible redundancy of gene functions during the evolution of the *GA20ox* gene family (Figure 4B).

The intraspecific and interspecific collinearity analysis showed that segmental duplication was the main reason for the expansion of the *GA20ox* gene family. During the expansion process, 80% of *PheGA20ox* genes had 12 and 13 homologous gene pairs with the monocotyledons rice and maize, respectively, while the bamboo species Ma bamboo had 30 homologous gene pairs. This indicated that most of the *PheGA20ox* genes were very conserved in evolution.

The identification of the genetic variation behind phenotypic differences between organisms and the evolutionary pressures that lead to change is one of the main goals of evolutionary biology [25,34]. Through selection pressure analysis, we were surprised to find positive selection pressure on 24% of *PheGA20ox*, which carry non-synonymous mutations that make Moso bamboo more adapted to the environment. Many positive selection pressures are rare in other phytohormone gene families. The large number of genes under positive selection pressure may suggest that the GA pathway plays an increasingly important role in the future development of Moso bamboo. Overall, although the *PheGA20ox* gene family is highly conserved within the family during the evolution of Moso bamboo, it shows an expansion trend in the whole gene family, and segmental duplication is the main reason for the expansion of the gene family.

Combining the results of intraspecific and interspecific collinearity analysis, phylogenetic analysis, and selection pressure analysis, we concluded that most of the genes of the *PheGA20ox* gene family of Moso bamboo showed conservative evolutionary relationships, and that a large number of homologous genes of the same rice gene existed in Moso bamboo. However, at the same time, the *PheGA20ox* family genes of Moso bamboo were again subjected to positive selection pressure compared with rice, which implied that there is a tendency for the family genes to expand in the future, and the functions of the family genes are likely to be further enriched. This was also corroborated by our analysis of gene expression patterns during the rapid growth of Moso bamboo, where no obvious differentially expressed genes were observed during the important rapid growth process of Moso bamboo, implying that possibly the functions of the relevant regulatory genes are likely to be further enriched in the future evolutionary process.

During Moso bamboo shoot development, *PheGA20ox* is involved in all stages of shoot growth and development, from shoot to adult bamboo, and *PheGA20ox3*, *PheGA20ox4*, *PheGA20ox5,* and *PheGA20ox6* are highly expressed in the winter shoot, suggesting that these four *PheGA20ox* genes may function mainly in the winter shoot period. We observed that 90% of the *PheGA20ox* genes were not significantly upregulated during the rapid growth stages of Moso bamboo shoots (from 300 to 900 cm in height), which may be partly owing to the fact that we did not detect the GA synthesis site during the rapid growth process of Moso bamboo, and partly because GA may have finished most of the accumulation process during the pre-growth stage of Moso bamboo, and the rapid growth period mainly relies on the source pool transport process to regulate the internode growth, which was also preliminarily confirmed in our expression pattern analysis. Meanwhile, the above inference still needs much experimental work to support it. The analysis of the evolutionary pattern of *PheGA20ox* family genes confirmed the overall expansion trend of *PheGA20ox* family genes in Moso bamboo, and in the process of evolution, it is possible that GA regulation during the rapid growth of Moso bamboo may play a more important role in the future. Miraculously, *PheGA20ox* genes are weakly expressed in the underground organs, unlike in Arabidopsis, where *GA20ox* is highly expressed in the roots [35]. The Moso bamboo *PheGA20ox* genes are mainly expressed in the floral organs, where they may have been primarily in GA biosynthesis.

GA usually crosstalks with other phytohormones to regulate plant growth and development. Analysis of promoter cis-acting elements showed that *PheGA20ox* family genes responded to abscisic acid, MeJA, salicylic acid, GA, and auxin elements, which was consistent with previous studies [36,37,38]. A regulatory relationship between *GA20ox* and *ARF* was indeed found in Arabidopsis, poplar, and tomato [18,19,20]. In Moso bamboo, transcriptome data analysis showed that *PheGA20ox3* and *PheGA20ox6* respond to GA and NAA, suggesting that *PheGA20ox3* and *PheGA20ox6* may be key genes in response to phytohormone crosstalk. This was demonstrated in yeast one-hybrid and dual luciferase experiments, where PheARF47, an upstream regulating factor of *PheGA20ox3* and *PheGA20ox6*, can bind the cis-P box element (a GA-binding element) and the cis-AuxRR element (auxin-binding element) of *PheGA20ox3*. It has been shown that ARF is widely involved in the regulation of auxin and other phytohormones. Combined with the results of our experiments, we have reason to believe that ARF also plays an important role in the integration of phytohormones in Moso bamboo, and it is important to clarify the important biological functions of ARF in order to investigate the multi-hormone crosstalk in Moso bamboo to regulate its rapid growth.

## 4. Materials and Methods

### 4.1. Gene Family Identification and Physicochemical Property Analysis

The Arabidopsis (version 11) and rice (IRGSP-1.0) *GA20ox* family members were obtained from the Arabidopsis genome database (http://www.arabidopsis.org/, accessed on 21 February 2023) and the rice genome database (http://rice.plant biology.msu.edu/index, accessed on 21 February 2023). The Moso bamboo *GA20ox* gene family members were determined by the local BLAST method (E-value = 1 × 10^−5^). The sequence information was further validated by conserved domain analysis in the NCBI database (https://www.ncbi.nlm.nih.gov/Structure/cdd/wrpsb.cgi, accessed on 21 February 2023) and Hidden Markov Model (HMM) (multiple sequence alignment method). Physicochemical properties of the proteins were obtained via the online website ExPASY (https://www.expasy.org/tools, accessed on 21 February 2023).

### 4.2. Analysis of Evolutionary Patterns

Chromosome distribution, genome replication analysis, interspecies collinearity analysis, and selection pressure analysis of Moso bamboo *GA20ox* gene family members were performed using TBtools software [39], mainly the Advanced Circos, One Step MCScanX, and Simple Ka/KS Calculator programs.

### 4.3. Phylogenetic Tree Construction

The protein sequences of *GA20ox* gene family members of other species were obtained from the Phytozome website [40], and the maximum likelihood phylogenetic tree was constructed using the TBtools software one-step build an ML tree program, with the spreading value set to 5000. Phylogenetic tree landscaping was conducted through the online website ChiPlot [41].

### 4.4. Transcriptome Data Acquisition

Transcriptome data were obtained from the NCBI GEO database (https://www.ncbi.nlm.nih.gov/geo/, accessed on 23 February 2023), index numbers GSE104596; GSE100172; GSE90517 [42,43,44]. The RPKM values of gene expression were used to analyze the expression levels of genes. For statistical purposes, the log2 of each expression was taken and the gene expression heat map was plotted using TBtools.

### 4.5. Promoter Analysis

The 2000 bp sequence upstream of the gene ATG was extracted as the promoter region by TBtools software. All promoters were pooled and cis-acting element prediction was performed through the PlantCARE cis-acting element website [45], and the cis-acting elements obtained were collated and visualized by the Gene Structure View (Advanced) program of TBtools software.

### 4.6. Yeast One-Hybrid Assay

The promoter regions of the *PheGA20ox3* and *PheGA20ox6* genes were cloned and ligated to the pHIS2 vector by seamless cloning. The yeast library retained in the laboratory was screened and the full-length CDS sequence of the obtained *PheARF47* target gene was ligated to the pGADT7 vector, while the cis-P box element (CCTTTTG) and cis-AuxRR element (GGTCCAT) were cloned (tandem three times) and then ligated to the pHIS2 vector to identify the PheARF47 binding element by yeast one-hybrid. The yeast one-hybrid procedure was performed according to the Clontech yeast one-hybrid system instructions (630491).

### 4.7. Dual Luciferase Reporter Assay

The CDS of the *PheARF47* gene was cloned into the pGreenII 62-SK vector and the promoter regions of the *PheGA20ox3* and *PheGA20ox6* were introduced into the pGreenII 0800-LUC vector. The constructed vectors were separately transformed into strain GV3101 (pMP90). The Agrobacterium strains containing the vectors were mixed and co-infested in tobacco leaves for transient expression. The luminescence ratio of firefly LUC to Renilla LUC was measured using a dual luciferase reporter system (Promega; catalog number: E1910) according to the manufacturer’s instructions. Experiments were repeated at least three times and results were expressed as mean ± standard deviation. Statistics were determined by using a *t* test (** represents *p* < 0.01, * represents *p* < 0.05).

## Data Availability

The original contributions presented in the study are included in the article/Supplementary Material, further inquiries can be directed to the corresponding author.

## Supplementary Materials

The following supporting information can be downloaded at: https://www.mdpi.com/article/10.3390/plants12152842/s1, Table S1: Identification of *GA20ox* family genes and physicochemical property analysis of Moso bamboo, Table S2: Genetic information on the syntenic relationships of Moso bamboo and other species, Table S3: Specific information on Moso bamboo and rice selection pressure analysis, Table S4: Analysis of the cis-acting elements of the *GA20ox* gene family promoter in Moso bamboo, Table S5: The information on the *ARF* gene family of Moso bamboo.
